# Endoscopist-Directed Propofol as an Adjunct to Standard Sedation: A Canadian Experience

**DOI:** 10.1093/jcag/gwz011

**Published:** 2019-04-26

**Authors:** Valérie Heron, Charlotte Golden, Seymour Blum, Gad Friedman, Polymnia Galiatsatos, Nir Hilzenrat, Barry L Stein, Andrew Szilagyi, Jonathan Wyse, Robert Battat, Albert Cohen

**Affiliations:** 1 Division of Gastroenterology, McGill University Health Center, Montreal, Quebec, Canada; 2 Division of Gastroenterology, SMBD Jewish General Hospital, Montreal, Quebec, Canada; 3 Department of Surgery, McGill University Health Centre, Montreal, Quebec, Canada

**Keywords:** Endoscopist-directed propofol, Endoscopy, Outcomes, Safety, Standard sedation

## Abstract

**Background:**

Sedation practices vary widely by region. In Canada, endoscopist-directed administration of a combination of fentanyl and midazolam is standard practice. A minority of cases are performed with propofol.

**Aims:**

To describe the safety of nonanaesthetist administered low-dose propofol as an adjunct to standard sedation.

**Methods:**

This was a single-centre retrospective study of patients having undergone endoscopic procedures with propofol sedation between 2004 and 2012 in a teaching hospital in Montreal. Procedures were performed by gastroenterologists trained in Advanced Cardiovascular Life Support. Sedation was administered by intravenous bolus by a registered nurse, under the direction of the endoscopist. Outcomes of procedures were collected in the context of a retrospective chart review using the hospital’s endoscopy database.

**Results:**

Of patients undergoing endoscopies at our centre, 4930 patients received propofol as an adjunct to standard sedation with fentanyl and midazolam. Cecal intubation rate for colonoscopies (*n* = 2921) was 92.0%. Gastroscopies (*n* = 1614), flexible sigmoidoscopies (*n* = 28), endoscopic retrograde cholangiopancreatography (*n* = 331) and percutaneous endoscopic gastrostomy insertion (*n* = 36) had success rates, defined as successful completion of the procedure within anatomical limits, of 99.0, 96.4, 94.0 and 91.7%, respectively. The average dose of propofol used for each procedure was 34.5 ± 20.8 mg. Fentanyl was used in 67.4% of procedures at an average dose of 94.3 ± 17.5 mcg. Midazolam was used in 92.7% of cases at an average dose of 3.0 ± 0.7 mg. Reversal agents (naloxone or flumazenil) were used in 0.43% of the cases (*n* = 21). Patients who received propofol were discharged uneventfully within the usual postprocedure recovery time. One patient required sedation-related hospitalization. For patients having received propofol in addition to standard sedation agents, 99.6% experienced no adverse events. There were no mortalities.

**Conclusion:**

The use of low-dose propofol as an adjunct to fentanyl and midazolam, administered by a registered nurse under the direction of the endoscopist was safe and effective in patients at our centre.

## INTRODUCTION

Sedation during endoscopy improves the quality of the examination by increasing patient satisfaction and decreases the rate of incomplete procedures (1,2). However, with several pharmacological agents available, sedation practices vary widely by region. In Canada, endoscopist-directed administration of a combination of an opioid and a benzodiazepine (commonly fentanyl and midazolam) is standard practice, while only a minority of cases are performed with propofol. In a recent Canadian survey, only 13% of adult gastroenterologists reported using propofol in routine colonoscopies, usually administered by an anaesthesiologist (3).

Fentanyl and midazolam both have a rapid onset of action and a relatively short half-life. In most patients, the use of these drugs safely and effectively relieves discomfort and anxiety related to the procedure. However, certain patients may be refractory to these medications or particularly intolerant to endoscopic procedures. In these cases, the use of low-dose propofol as an adjunct to standard agents allows for more effective sedation without prolonging recovery times (4,5).

Endoscopist-directed propofol administration in both upper and lower endoscopy has been demonstrated to be safe and effective (6–8). However, controversy surrounding its routine use remains. The use of propofol by trained endoscopists in select patients has been supported by several societies including the American College of Gastroenterology (ACG), American Gastroenterological Association (AGA), American Society for Gastrointestinal Endoscopy and Canadian Association of Gastroenterology (CAG) (9–11). Despite this, many gastroenterologists are hesitant to use propofol for sedation. This is likely due in part to current Food and Drug Administration labelling which warns that propofol “should be administered only by persons trained in the administration of general anesthesia” (12). This perception that the use of propofol should be reserved for anaesthesiology specialists, as well as local or institutional restrictions have limited its use in routine cases. In the present study, we aimed to describe the safety and efficacy of low-dose endoscopist-directed propofol in combination with standard sedation agents in a Canadian teaching hospital.

## METHODS

### Study Design and Population

This was a single-centre retrospective study of 4930 patients having undergone gastroscopy, colonoscopy, flexible sigmoidoscopy, endoscopic retrograde cholangiopancreatography (ERCP) or percutaneous endoscopic gastrostomy (PEG) insertion between 2004 and 2012 at the Sir Mortimer B Davis, Jewish General Hospital, Montreal, Canada. Endoscopic procedures during which propofol was used were identified using the hospital’s electronic medical records (Chartmaxx, Quest Diagnostics Inc., Secaucus, NJ and endoscopy records (Endovault, EndoSoft, LLC, Schenectady, NY). Cases were excluded if propofol was administered by an anaesthetist or as an intravenous (IV) infusion in an intensive care unit (ICU) or emergency room setting. Endoscopic procedures were performed by seven gastroenterologists, each trained and certified in Advanced Cardiovascular Life Support (ACLS). In all cases, patients provided written consent for the procedure and sedation. Sedation was administered on a voluntary basis according to patient preference. The type of sedative was chosen at the discretion of the endoscopist. Factors influencing the decision to use propofol included patient discomfort despite standard sedation, previous difficulty with sedation and patient comorbidities. Propofol was administered largely as an adjunct to fentanyl and midazolam, alone or in combination, if the level of sedation–analgesia was insufficient. Sedative agents were administered by IV bolus by a registered nurse trained in endoscopy, under the direction and supervision of the endoscopist. The initial dose of propofol was a 20 mg bolus. Dosing was titrated to patient’s comfort with repeated boluses administered if needed usually of 10 mg at a time. The nursing staff was trained to recognize signs of over sedation and received Basic Cardiac Life support (BCLS) certification. The registered nurse administering sedation ensured ongoing monitoring of the patient’s blood pressure, heart rate, oxygen saturation and respiratory rate throughout the procedure, while a second nurse or technician offered technical assistance for the procedure. Patients routinely received supplemental oxygen by means of nasal cannula.

### Data Collection

Data from medical records including the type of procedure, whether the procedure was successfully completed, and the doses of sedative agents administered were collected. Procedures were considered to be successful if further advancement was limited by a mechanical obstruction rather than by patient comfort. Adverse events such as hypoxemia requiring mechanical ventilation, use of reversal agents (naloxone or flumazenil), hypotension requiring medical intervention, transfer to the ER or ICU and death were also recorded.

### Statistical Analyses

Mean sedative doses administered as well as rates of adverse events and rates of complete examinations were calculated using descriptive statistics. These outcomes were calculated for the overall study population as well as for each type of procedure performed.

## RESULTS

A total of 4930 patients were included in this study. The cecal intubation rate for colonoscopies (*n* = 2921) was 92.0%. Gastroscopies (*n* = 1614), flexible sigmoidoscopies (*n* = 28), ERCP (*n* = 331) and PEG insertion (*n* = 36) had success rates of 99.0, 96.4, 94.0 and 91.7%, respectively.

Propofol was used as monotherapy in a minority of cases (*n* = 271, 5.5%), usually in patients reporting prior intolerance to standard sedatives. Combinations of propofol and fentanyl and of propofol and midazolam were administered in 1.8% (*n* = 91) and 27.1% (*n* = 1336), respectively. Propofol, Fentanyl and midazolam were used in combination in 65.6% (*n* = 3232) of the cases.

The mean dose of propofol used for each procedure was 34.5 ± 20.8 mg. Propofol doses for each type of procedure are shown in Table 1. Mean doses of fentanyl and midazolam were 94.3 ± 17.5 mcg and 3.0 ± 0.7 mg, respectively.

**Table 1.** Mean doses of propofol used for each type of procedure

	Average dose of propofol IV ± standard deviation (mg)
Colonoscopy (*n* = 2921)	34.7 ± 18.2
ERCP (*n* = 331)	54.8 ± 41.0
Gastroscopy (*n* = 1614)	29.4 ± 14.7
PEG (*n* = 36)	52.9 ± 39.2
Sigmoidoscopy (*n* = 28)	35.5 ± 24.9
Total	34.5 ± 20.8

ERCP, endoscopic retrograde cholangiopancreatography; IV, intravenous; PEG, percutaneous endoscopic gastrostomy.

Reversal agents (naloxone or flumazenil) were used in 0.43% of the cases (*n* = 21). Patients having received reversal agents were discharged uneventfully within the usual postprocedure recovery time. One patient required transfer to the emergency department (0.02%). This was an 87-year-old male with decompensated cirrhosis, Wernicke’s encephalopathy and coronary artery disease who was undergoing gastroscopy for a massive upper gastrointestinal bleed. He deteriorated in the endoscopy unit after receiving 50 mcg of fentanyl and 10 mg of propofol. He underwent a repeat gastroscopy the following day which revealed a gastric ulcer.

Mean doses of propofol, fentanyl and midazolam administered in these 22 cases were 30 ± 12.0 mg, 95.2 ± 15.0 mcg and 3.2 ± 0.8 mg, respectively. Most patients having received propofol in addition to standard sedation experienced no adverse events (99.6%). There were no deaths or need for mechanical ventilation.

## Discussion

This large retrospective study supports the use of low-dose endoscopist-directed propofol as an adjunct to standard sedation in select patients undergoing routine endoscopy. The overall rate of adverse events was low (0.45%) and no serious complications such as mechanical ventilation or death occurred. These are the largest reported outcomes for endoscopist-directed propofol in Canada and the results are in keeping with numerous other studies demonstrating the safety of propofol sedation in gastrointestinal endoscopy (8). A recent meta-analysis showed no increase in the cardiopulmonary complications with propofol sedation compared with traditional sedatives during endoscopic procedures (pooled odds ratio [OR] 0.77; 95% confidence interval [CI]: 0.56–1.07) (6).

The use of propofol poses several advantages over other forms of sedation. Indeed, propofol possesses a favourable pharmacological profile with a rapid onset of action and a short half-life (13). When compared with midazolam, sedation with propofol has been associated with faster recovery and earlier discharge times (14). Compared to standard sedation, propofol has also been shown to improve patient cooperation (14,15). This in turn may increase the rate of successfully completed procedures. Our study showed a 92% cecal intubation rate for colonoscopies, meeting the 90% target recommended by current guidelines despite propofol being reserved primarily for patients with poor tolerance of endoscopic procedures (16).

The doses of propofol required for moderate sedation in endoscopy are much smaller than those typically used by anaesthesiologists for the induction of general anaesthesia (12). The mean dose of propofol in our study was 34.5 ± 20.8 mg. This dosage is in keeping with those reported in the current literature on endoscopist-directed propofol sedation (4).

Given the small dosage required and the extremely low complication rates reported, the routine presence of an anaesthesiologist for propofol sedation is unlikely to have a significant impact on safety and will certainly result in increased cost which is an important limitation in today’s health care system (17). However, it is crucial that the team responsible for sedation rigorously monitor the patient and be prepared to manage any foreseeable complications. In accordance with current guidelines, each endoscopist in our study had received ACLS training, and monitoring was ensured by a nurse dedicated solely to this task (4,10,11,18). Furthermore, prior to discharge from the recovery room, patients were required to meet set clinical criteria defined by the Pasero Opioid-induced Sedation Scale (POSS) (19,20).

The decision to administer propofol after failure of standard therapy was made by each endoscopist based on standard sedation risk assessment. Careful patient selection may also be beneficial to minimize complication rates. Anticipated duration and complexity of the procedure as well as patient characteristics such as age, American Society of Anaesthesiologists’ (ASA) classification, Mallampati score, and a history of obstructive sleep apnea should be taken into consideration when choosing the type and dose of sedative. In high risk cases, a consultation with an anaesthesiologist should be considered (11,21).

This study describes a unique experience among Canadian centres where, overall, propofol is seldom used. To our knowledge, low-dose endoscopist-directed propofol sedation as an adjunct to fentanyl and midazolam has never been studied in a population of this size in Canada. Furthermore, the size of our patient population is among the largest reported worldwide for a single centre. A limitation of our study is its retrospective observational design. Accurate assessment of adverse events was dependent on thorough documentation and on patients presenting back to our institution with complications. Furthermore, successful completion of procedures was the only surrogate available to assess effectiveness of propofol sedation. Though no control group was available to compare success rates among patients receiving other forms of sedation, the cecal intubation rate in this study met current quality standards (16). In future prospective studies, the use of a matched control group would help to more accurately compare efficacy and safety of propofol in combination with fentanyl and/or midazolam compared to standard sedation alone.

Overall, this study provides real-world Canadian data on the use of low-dose endoscopist-directed propofol as an adjunct to standard sedation in a hospital setting. With proper training and careful monitoring, propofol is a safe, effective and affordable in addition to standard sedation in selected patients. Further prospective studies are needed to better quantify the impact of propofol sedation on safety outcomes, completion rates and cost.

## Conflicts of Interest

The authors declare that there is no conflict of interest regarding the publication of this paper.

## Notes

An earlier version of this abstract was previously presented in poster form at the 2018 Canadian Digestive Disease Week and published in the *Journal of the Canadian Association of Gastroenterology*, Volume 1, supp 2, March 2018.
